# Evaluating the Research Productivity in Pulpotomy: A Bibliometric Analysis

**DOI:** 10.7759/cureus.72893

**Published:** 2024-11-02

**Authors:** Pillai Arun Gopinathan, Ikram UI Haq, Fares Almutairi, Feras Alsultan, Adeeb Alnajashi, Meshaal Alahmari, Bandar Altaweilae, Bijesh Yadav

**Affiliations:** 1 Department of Maxillofacial Surgery and Diagnostic Sciences, College of Dentistry, King Saud Bin Abdulaziz University for Health Sciences, King Abdullah International Medical Research Centre (KAIMRC), Ministry of National Guard Health Affairs, Riyadh, SAU; 2 College of Dentistry, King Saud Bin Abdulaziz University for Health Sciences, Riyadh, SAU; 3 College of Dentistry, College of Dentistry, King Saud Bin Abdulaziz University for Health Sciences, King Abdullah International Medical Research Centre (KAIMRC), Ministry of National Guard Health Affairs, Riyadh, SAU; 4 College of Dentistry, King Saud Bin Abdulaziz University for Health Sciences, King Abdullah International Medical Research Centre (KAIMRC), Ministry of National Guard Health Affairs, Riyadh, SAU; 5 Department of Biostatistics/Department of Population Health, Division of Biostatistics, King Abdullah International Medical Research Centre (KAIMRC), Riyadh, SAU

**Keywords:** bibliometric, citations, pediatric dentistry, pulpotomy, research productivity

## Abstract

The aim of this study was to appraise the bibliometric parameters of pulpotomy in the past 24 years from January 2000 to December 2023. The dataset was obtained from the Web of Science (WoS) database. The filter publication years were applied. The bibliographic details of all types of documents indexed in the WoS database under keywords of “*Dental pulp exposure*” or “*Coronal pulpotomy*” or “*Partial pulpotomy*” or “*Permanent pulpotomy*” or *Pulpectomy* published in the targeted period (2000-2023) were downloaded. The bibliometric characteristics such as the type of documents, growth of publications and citations by years, level of evidence, top publication channels, top countries, top contributing organizations, top authors, and keywords were analyzed. MS Excel, VOSviewer, and R were utilized for data analysis. A total of 738 documents were identified, and they were published in 213 sources contributed by 3,017 authors from 75 countries. A fluctuated growth was recorded and the majority of the research (82.12%) was published in the latter half (2012-2023) of the targeted period. Most of the studies utilized the Level of Evidence (LoE) IV but the studies on LoE-I gained the highest average citations. The majority of papers were produced by India, closely followed by Brazil and the United States. Thus, the upward trend in the latter half was suggestive of advancement in oral health education along with enhancement in research publications.

## Introduction and background

Dental pulp inflammation is treated with a critical dental procedure known as a pulpotomy, particularly in primary teeth that have been damaged or have caries [1]. In this process, the diseased or inflamed pulp tissue inside the tooth's crown is surgically removed, leaving the healthy pulp inside the root canals intact. Pulpotomy is mostly used in pediatric dentistry, which involves partially removing the tooth pulp to maintain the vitality of the pulp tissue. The objectives of this treatment are to reduce discomfort, stop further infection, and keep the damaged tooth functional [2,3]. Pulpotomy has garnered significant attention from researchers and clinicians due to its use in the treatment of dental caries and pulp disorders [4,5].

Bibliometric analysis assists as an influential tool to assess scholarly literature on a specific topic, permitting researchers to recognize the prevailing research trends, evaluate the impact of scientific research, and reveal knowledge gaps [6,7]. By systematically examining published works, citation metrics, and authorship patterns, bibliometric findings can offer valuable insights into the development of specific dental themes, the development of techniques, and the efficiency of various materials used in the procedure [8]. Bibliometric analysis can furnish information on productivity rates and publication trends and also offer statistical analyses. It is additionally employed to examine the productivity of the researcher, organizations, journal trends, nations pertaining to a specific subject, and research patterns throughout diverse disciplines of study [9]. Understanding the scholarly landscape of dental literature through bibliometric examination is vital for several reasons. It highlights the publication growth and quality of research conducted over time, disclosing shifts in focus areas, methodologies, and outcomes [10]. It categorizes key contributors and institutions leading the field, which can substitute collaboration and knowledge sharing and this analysis also can inform future research directions, guiding investigators toward underexplored aspects of dentistry and enhancing clinical practice [11,12].

A bibliometric review of the 100 most cited articles on endodontic therapy in primary teeth revealed that pulpotomy was found the most used keyword. These articles were published from 1964 to 2017 and 84% of the articles were published after 1999. The authors from 24 countries contributed, but the United States produced 21 articles followed by Brazil and Turkey. University of São Paulo emerged as the most contributing institution, while Pediatric Dentistry Journal and International Endodontic Journal published 16 and 15 articles, respectively [13]. Another study analyzed the 1,197 papers on vital pulp therapy. A slow progress (39.51%) was observed from the first six decades (1946-2004) and an exponential growth of publication was recorded after 2012. The authors from the United States, Iran, and Brazil contributed most of the papers. Out of 176 journals, Journal of Endodontics secured the top ranked with 116 papers, followed by Pediatric Dentistry and International Endodontic Journal with 77 and 67 papers, respectively. “Pulpotomy” was found to be the most occurred keyword than “mineral trioxide aggregate” [14].

Sousa et al. carried out the bibliometric analysis of the 50 most cited articles on vital pulp therapies. These articles were cited 1,905 times (38.10 cites/article). “Mineral trioxide aggregate” and “vital pulp therapy” were found to be the top keywords. Most of the articles were produced by the United States followed by Ireland and the highest number of articles were published in the Journal of Endodontics and International Endodontic Journal [12]. Another study examined the 100 most cited articles on pediatric dentistry. Most of the articles opted for the literature review and cross-sectional study designs. Caries and Pulp therapy were the topmost research themes while the United States produced 43% of the articles. About three-fourths (n=72) of articles were published in two journals, Paediatric Dentistry and International Journal of Paediatric Dentistry [15]. Ohta et al. examined the 1,311 articles on pediatric dentistry published in 20 years (1999-2018) and stated that more than half of the literature was contributed by the United States, England, and Brazil. Cardiology and restorative dentistry were found to be the preferred areas of research [8]. A recent bibliometric study on pediatric dental sedation reveals that there has been a notable increase in the amount of research on this topic over the past three decades (1993-2022). The United States has made a significant contribution, and the majority of the research focused on sedation-related side effects and the remedies for them [16].

Pulpotomy is one of the important procedures done for pediatric patients to preserve tooth vitality. There is a clear scarcity of thorough bibliometric analyses that demonstrate the notable properties of research scenarios and new approaches in pulpotomy research. It is imperative to fill this knowledge gap to guide future research efforts in various communities and to improve clinical practices. This bibliometric analysis aims to comprehensively review the current state of research on pulpotomy, exploring publication trends, citation impacts, and emerging themes within the literature. By synthesizing this information, we hope to contribute to a better understanding of pulpotomy's role in modern dentistry and to identify opportunities for future inquiry.

## Review

Study design

This study was intended to evaluate the progress of research in pulpotomy and utilized a bibliometric research approach to analyze the publication network. The institutional review board (IRB) or ethical approval was not required for bibliometric study as it uses publicly available data.

Search strategy

A quantitative bibliometric investigation on the retrieved dataset, data was searched from the Web of Science (WoS) database using the following search terms on October 10, 2024: “Dental pulp exposure” (All Fields) or “Coronal pulpotomy” (All Fields) or “Partial pulpotomy” (All Fields) or “Permanent pulpotomy” (All Fields) or Pulpectomy. 

**Figure 1:**
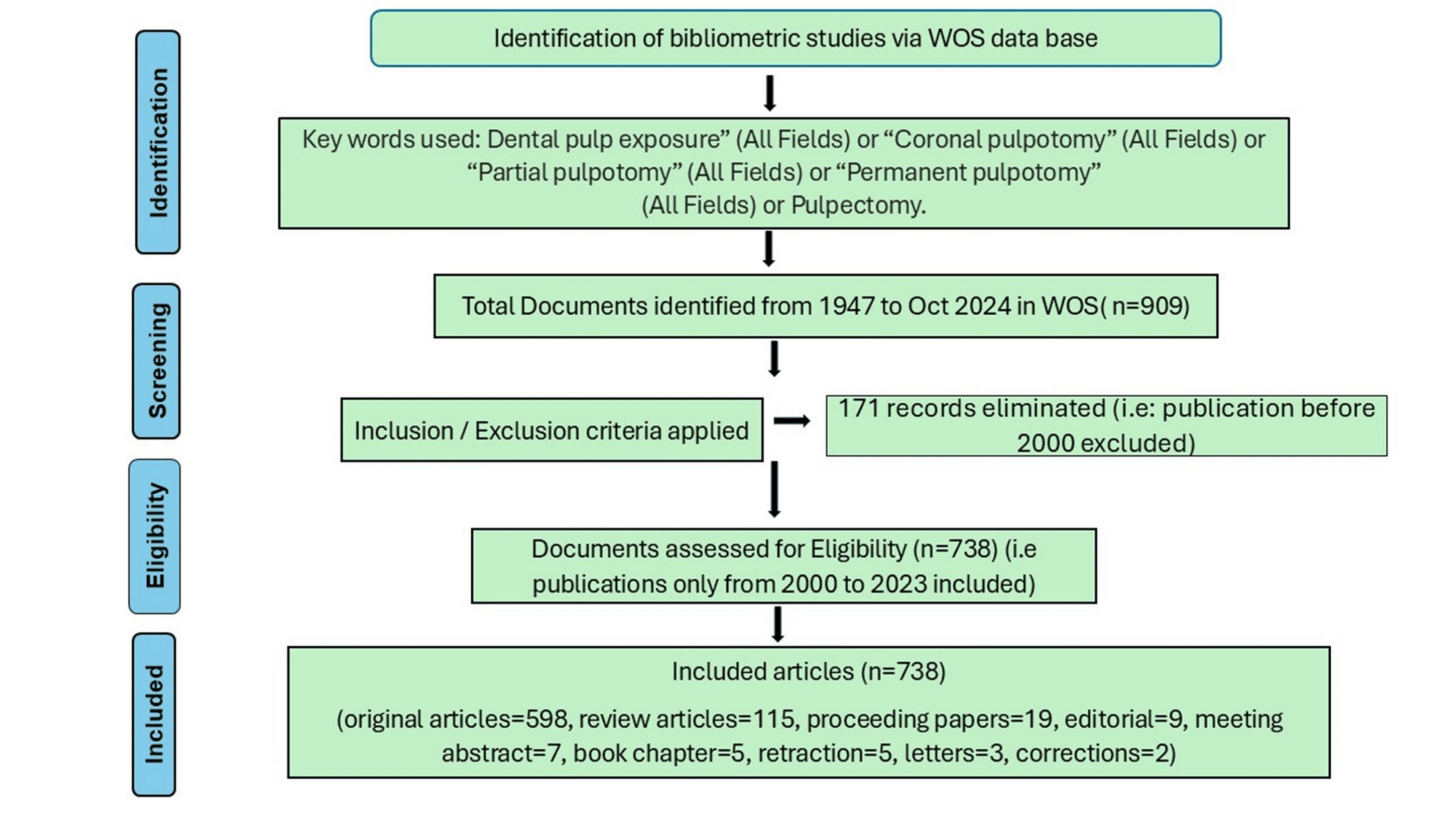
Methodology flow chart

Firstly, we obtained a dataset of 909 documents, published between 1947 and October 10, 2024. Then, we implemented the inclusion/exclusion criteria, choosing the years of publication from 2000 to 2023, and eliminating the publication records that had been published prior to the year 2000. We did not include the records for 2024, as the year is still in progress. We didn't use any further filters. We eliminated the 171 records, leaving 738 papers up for quantitative analysis. The flowchart is shown in Figure 1.

Microsoft Excel (v.16, Microsoft Corporation, Redmond, Washington), and VOSviewer (v.1.6.10) software were used for the data visualization and analysis. The study assessed vital bibliometric indicators, such as the examination of publications, the year of publication, the frequency of citations, publishing sources, top countries, institutions, authors, authorship patterns, keywords, and the characteristics of the top 10 highly cited papers.

Statistical analysis

The proportion of the comparison of open-accessed documents vs. subscription-based documents, documents published in dental vs. non-dental journals and clinical vs. non-clinical journals was done by using the chi-square test. The results were presented as proportion difference with 95% CI and p<0.05 was considered as significant. The comparison was made by using R 4.4 software (R Foundation for Statistical Computing, Vienna, Austria).

Results

Types of Documents

The examination of types of documents revealed that out of 738 total documents, original research articles (n=598; 81%) were a major part, followed by review articles (n=115; 15.58%), and proceeding papers (n=19; 2.57%). The other documents comprised editorial materials (n=9), meeting abstract (n=7), book chapters (n=5), retracted publications (n=5), letters (n=3) and two corrections. 

Comparisons of Papers Based on Accessibility, Publication Sources and Nature of Study

Table 1 presents the analysis of different types of papers based on accessibility, publication sources and nature of study focusing on citation impact. Subscription-based papers have higher average citations (20.11 cites/paper) than open-access papers (17.04 cites/paper). The proportion of citation difference between open-access and subscription-based was 3.07 (95% CI: 1.76 - 4.38) and this was statistically significant (p<0.001). This advocates that while open access increases accessibility, subscription-based papers may be cited more frequently. Papers published in dental journals significantly outperform those in non-dental journals in both total citations and citation impact. This indicates that research published in dental journals is more influential and recognized within the dental academic community. There was about 10% (95% CI: 8.6% - 11.5%) significance difference of publication in this type (p=0.009). Non-clinical studies have a higher citation impact than clinical studies, which could propose that findings in non-clinical research are being cited more frequently. There was 6.7% (95% CI: 5.5% - 8.14%) significance difference of citation in these journals (p=0.007).

**Table 1:** Distribution of papers by accessibility means, publication sources and nature of study

Variable	Accessibility/ Dental and Non-dental Sources	Total Papers	Total Citations	Citation Impact	Difference (95% CI)	p-value
Means of Accessibility	Open-Accessed Documents	329	5605	17.04	3.07(1.76 - 4.38)	<0.001
	Subscription-Based Documents	409	8226	20.11		
Publication Sources	Documents Published in Dental Journals	519	11279	21.73	10.08(8.62-11.54)	0.009
	Documents Published in Non-dental Journals	219	2552	11.65		
Nature of Study	Clinical Studies	448	7197	16.06	6.82(5.5-8.14)	0.007
	Non-clinical Studies	290	6634	22.88		

Analysis of Levels of Evidence (LoEs)

**Figure 2:**
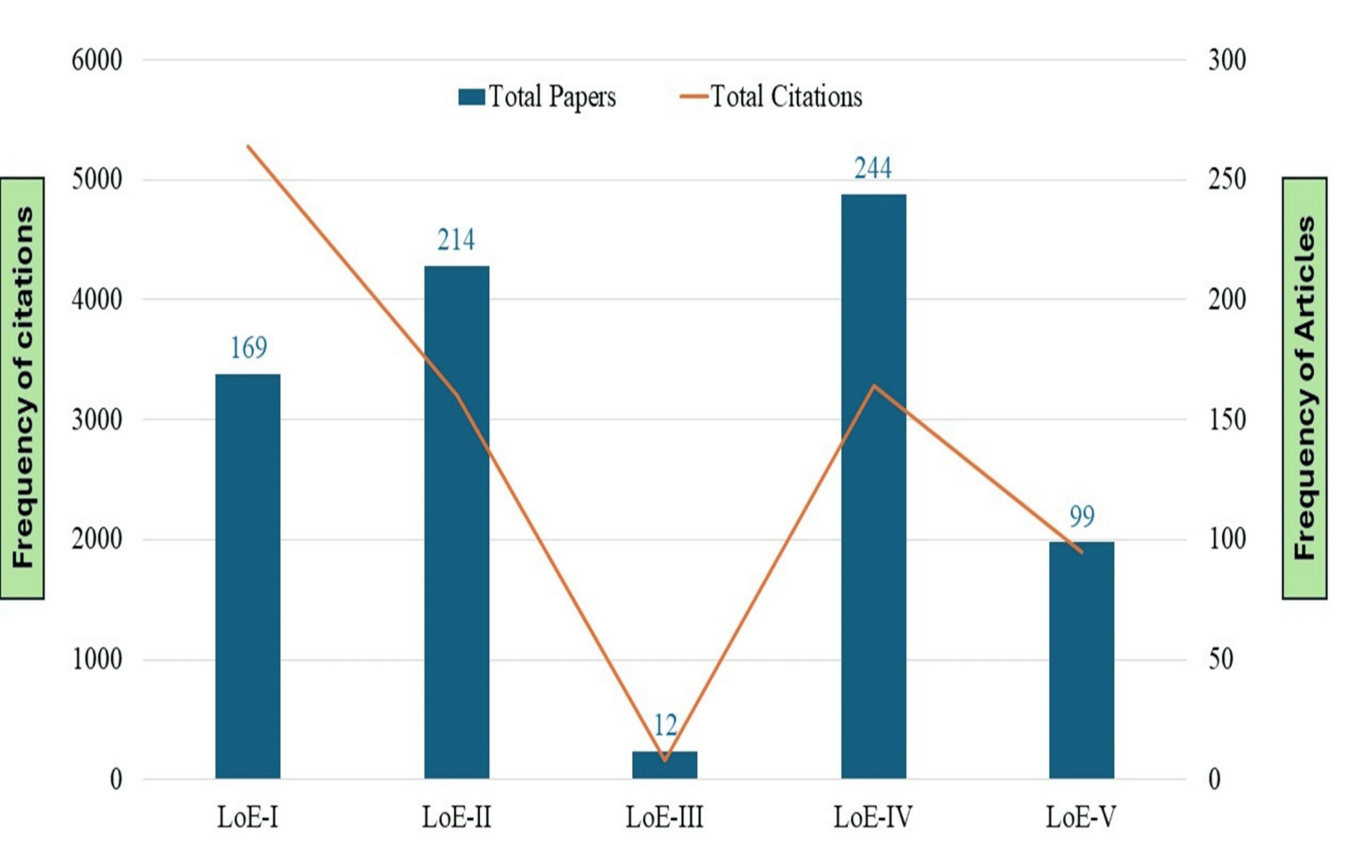
Frequency of level of evidence in pulpotomy LoE: Level of evidence

The analysis of LoEs shown in Figure 2 described that one-third of the papers (n=244; 33.06%) belonged to LoE-IV, followed by LoE-II (n=214; 29%), and LoE-I (n=169; 22.90%). The studies related to LoE-I gained the maximum citation impact (31.32 cites/paper), followed by LoE-V (19.16 cites/paper). The investigation of the LoE data reveals significant trends in citation impact, emphasizing the importance of high-quality research. While the importance is rightly placed on generating vigorous evidence, lower LoEs also play a crucial role in the research network.

Evolution of the Literature on Pulpotomy and Its Impact

**Figure 3:**
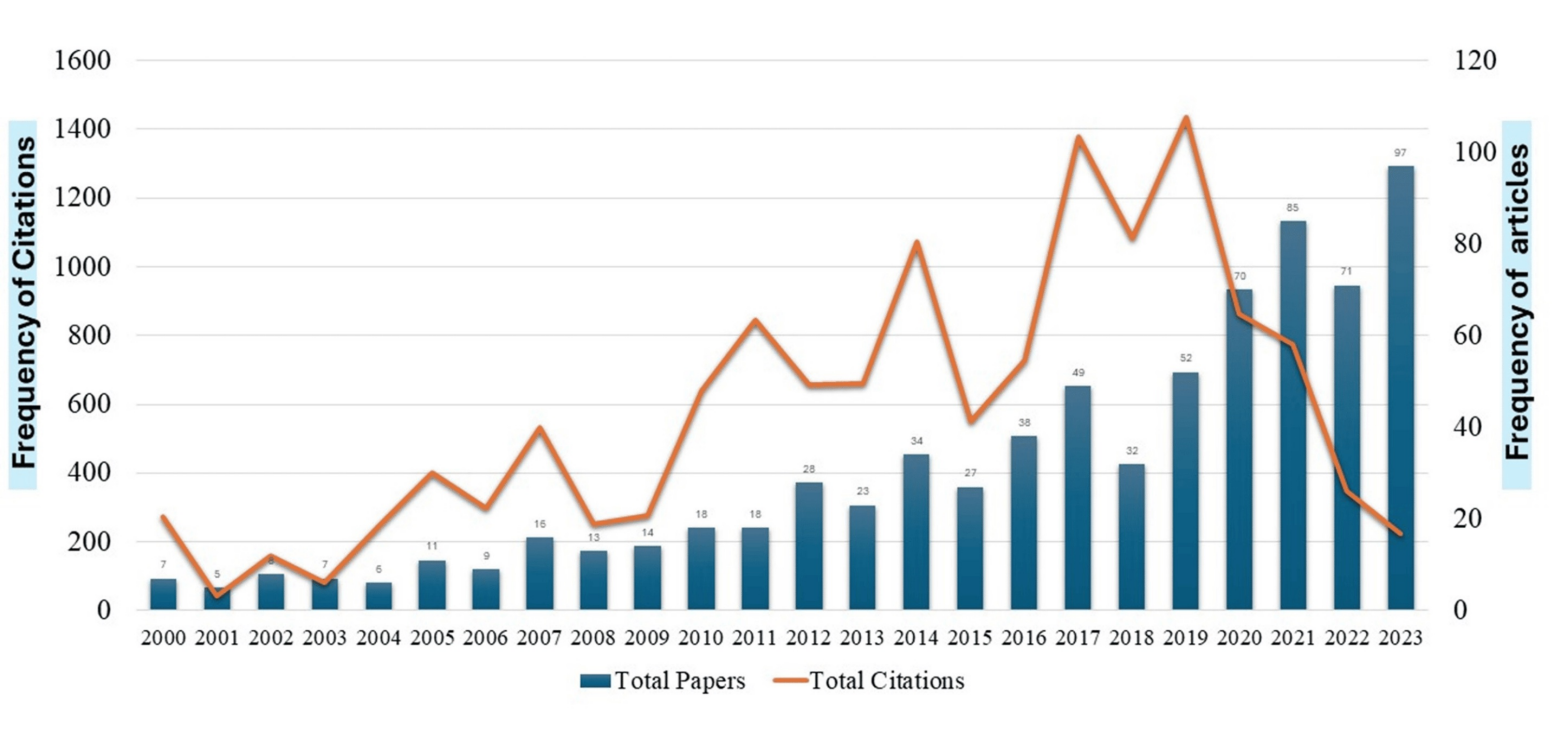
Growth of papers and citations by year

A total of 738 papers have been selected on pulpotomy from the WoS database which was published between the years 2000 and 2023. Figure 3 displays that a sluggish research growth (n=132; 17.88%) was found during the first 12 years (2000-2011) with an average of 11 papers per year but remarkable growth (n=606; 82.12%) was observed in the last 12 years (2012-2023), with an average of 50.50 papers per year. 

There is a vibrant rising inclination in the growth of papers after 2012, with a substantial increase in 2020 and 2021. The highest number of papers (n=97) was published in 2023. All these papers (n=738) were cited 13,831 times, with a mean ratio of 18.74 cites/paper. The papers published in the first 12 years gained 30.71 cites/paper, while the papers published in the last 12 years received 16.13 cites/paper. The number of citations generally increased over the years. Citation impact, which is calculated as total citations divided by total papers, shows a fluctuating trend. The highest citation impact was gained by the papers published in 2011 and 2004 with 47 and 41 cites/paper, respectively.

Frequently Used Publication Sources

The selected papers (n=738) have been published in 213 different sources, more than half of the sources (n=119; 55.86%) had published one paper, and 40% (n=296) of the papers have been published in top 10 journals (Table 2) and these journals gained 8,134 citations with an average of 27.47 cites/paper. Journal of Endodontics leads with 73 papers, followed by International Endodontics Journal and Journal of Clinical Pediatric Dentistry with 48 and 34 papers, respectively. The impact factor of the journal has been measured by the average number of citations to papers published in the journal during the last two years. The impact factor of the journals has been mentioned in the Journal Citation Report (JCR) of the Web of Science. The study followed the JCR 2023 edition for recording the impact factor of journals. Journal of Dental Research has the highest impact factor (5.7) and a very high citation impact (58.36), representing that its papers are extensively cited and qualified for their volume, followed by International Endodontics Journal (5.4) also displaying strong citation impact (42.29), reflecting its impact in the field. 

**Table 2:** Top 10 most frequently used sources of publications

Serial No.	Name of Journal	Impact Factor JCR 2023	Total Papers	Total Citations	Citation Impact
1.	Journal of Endodontics	3.5	73	3257	44.62
2.	International Endodontics Journal	5.4	48	2030	42.29
3.	Journal of Clinical Pediatric Dentistry	1.5	34	309	9.09
4.	European Archives of Paediatric Dentistry	2.3	27	506	18.74
5.	Pediatric Dentistry	1.5	26	516	19.85
6.	International Journal of Paediatric Dentistry	2.3	22	202	9.18
7.	Journal of Clinical and Diagnostic Research	0.2	18	132	7.33
8.	Clinical Oral Investigations	3.1	17	321	18.88
9.	Pediatric Dental Journal	0.6	17	44	2.59
10.	Journal of Dental Research	5.7	14	817	58.36

Top 10 Countries

The authors belonged to 75 countries, contributing to 738 papers, and the details of the top 10 productive countries with papers, citations and citation impact are shown in Table 3. The authors from India contributed the maximum papers (n=117), followed closely by Brazil (n=101) and the United States (n=98). England has the highest average citations at 45.04 cites/paper, signifying that its papers are very influential despite the fairly lower quantity (n=24). In contrast, India has a lower citation impact (7.5 cites/paper), representing that while it published a lot of research, the average paper does not gain many citations.

**Table 3:** Top 10 most productive countries with publication details

Serial No.	Name of Country	Total Papers	Total Citations	Citation Impact
1.	India	117	878	7.5
2.	Brazil	101	1645	16.29
3.	United States	98	3296	33.63
4.	Japan	61	1609	26.38
5.	Iran	44	1051	23.89
6.	China	44	878	19.95
7.	Turkey	33	440	13.33
8.	France	25	950	38.00
9.	England	24	1081	45.04
10.	Saudi Arabia	24	339	14.13

Co-authorship Network

**Figure 4:**
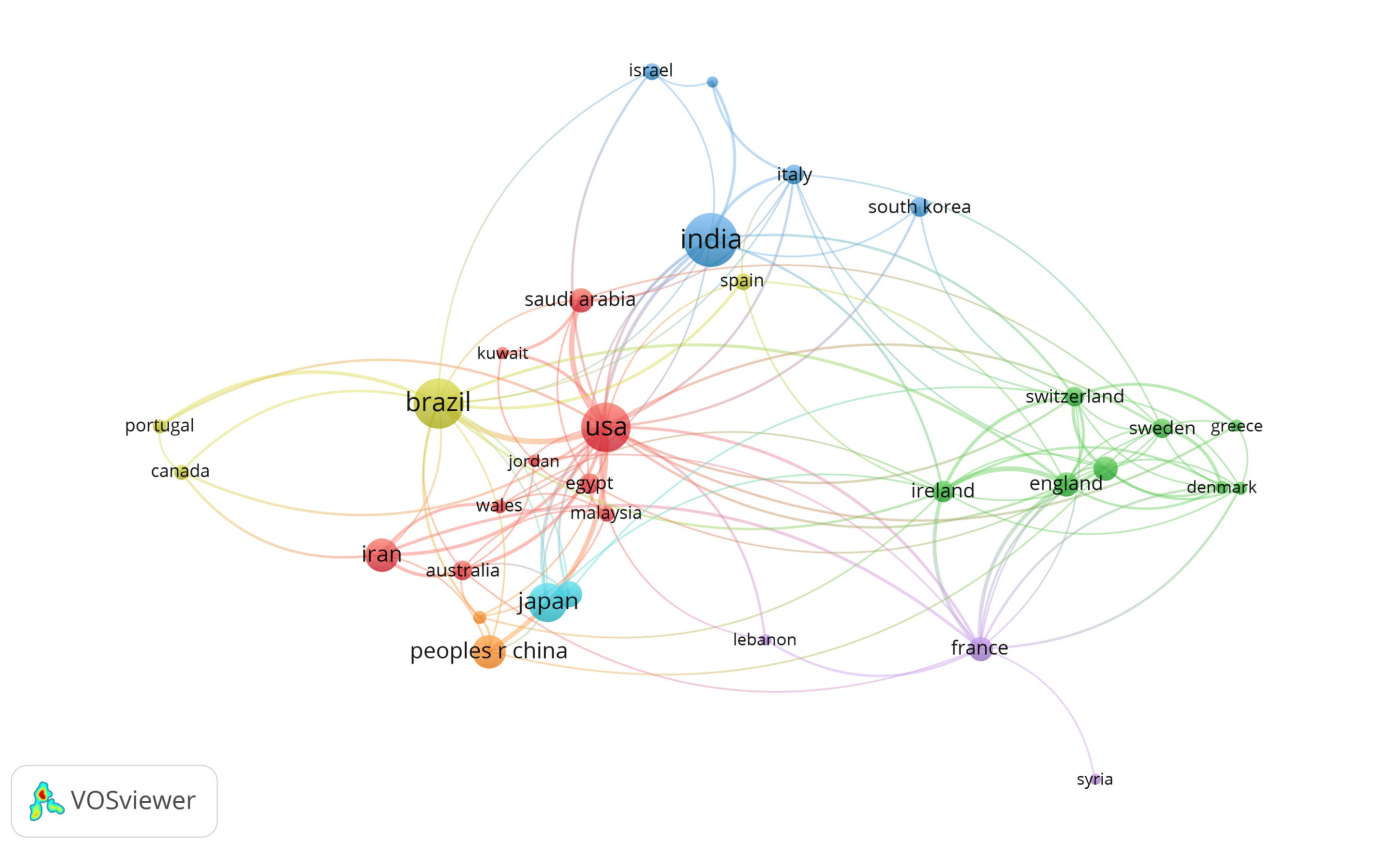
Co-authorship network of countries

The analysis co-authorship network of countries has been performed through VOSviewer software. Out of total 75 contributing countries, 33 countries met the threshold with at least five papers, connected with each other in seven clusters. There are nine countries in the topmost cluster (Australia, Egypt, Iran, Jordan, Kuwait, Malaysia, Saudi Arabia, United States, and Wales), eight in the second cluster (Denmark, England, Germany, Greece, Ireland, Norway, Sweden, and Switzerland) and five countries in the third cluster (India, Indonesia, Israel, Italy, and South Korea) as shown in Figure 4.

Top Contributor Institutions

Authors affiliated with 849 institutions contributed to 738 papers, 616 (72.55%) institutions contributed to a single paper, and 233 (27.45%) institutions produced more than one paper each. The publication output and citation impact of the top 10 institutions are shown in Table 4. Saveetha Dental College Hospital (n=31) and Universidade de Sao Paulo (n=23) show higher total outputs but relatively lower citation impacts, while Aichi Gakuin University has the highest citation impact (84.27 cites/paper), indicating that its research is highly influential despite a lower output. National Center for Geriatrics Gerontology follows closely (74.82 cites/paper), displaying promising research progress and recognition. Trinity College Dublin produced 18 papers with a high citation impact (45.56 cites/paper), reflecting a solid balance between quantity and quality.

**Table 4:** Top 10 institutions

Serial No.	Name of Institution	Total Papers	Total Citations	Citation Impact
1.	Saveetha Dental College Hospital, India	31	284	9.16
2.	Universidade de Sao Paulo, Brazil	23	298	12.96
3.	Trinity College Dublin, Ireland	18	820	45.56
4.	Egyptian Knowledge Bank, Egypt	17	159	9.35
5.	National Center for Geriatrics Gerontology, Japan	17	1272	74.82
6.	Universidade Federal Do Rio De Janeiro, Brazil	17	211	12.41
7.	Universidade Estadual Paulista, Brazil	14	383	27.36
8.	Islamic Azad University, Iran	12	326	27.17
9.	Aichi Gakuin University, Japan	11	927	84.27
10.	Chulalongkorn University, Thailand	11	542	49.27

Most Prolific Authors

A total of 3,017 authors contributed to 738 papers, 88% (n=2,656) of the authors had contributed to a single paper and a small group of authors (n=361; 12%) contributed to more than one paper. The publication record of the top 10 authors is demonstrated in Table 5. Masashi Murakami has the highest citation impact (92.43 cites/paper) with just seven papers, demonstrating that his research has been vastly influential, followed by Misako Nakashima with a strong citation impact (79.06 cites/paper) from 16 papers. Henry Duncan and Koichiro Iohara show a decent stability of research productivity with 15 and 14 papers, respectively, with 53.80 and 77.14 cites/paper.

**Table 5:** Top 10 authors

Serial No.	Name of Author	Affiliation	Total Papers	Total Citations	Citation Impact
1.	Laura Primo	Universidade Federal Do Rio De Janeiro, Brazil	18	211	11.72
2.	Misako Nakashima	National Center for Geriatrics Gerontology, Japan	16	1265	79.06
3.	Henry Duncan	Trinity College Dublin, Ireland	15	807	53.80
4.	Ganesh Jeevanandan	Saveetha Dental College Hospital, India	14	241	17.21
5.	Koichiro Iohara	National Center for Geriatrics Gerontology, Japan	14	1080	77.14
6.	Roberta Barcelos	Universidade Federal Do Rio De Janeiro, Brazil	11	166	15.09
7.	Helena Fransson	Malmö University, Sweden	9	671	74.56
8.	Saeed Asgary	Shahid Beheshti University Of Medical Sciences, Iran	8	197	24.63
9.	Amaury Pozos-Guillen	Autonomous University of San Luis Potosí, Mexico	8	86	10.75
10.	Masashi Murakami	National Center for Geriatrics Gerontology, Japan	7	647	92.43

Laura Primo and Roberta Barcelos from Brazil have comparatively lower citation impacts (11.72 and 15.09, respectively) despite a reasonable number of papers (18 and 11). Several authors from Japan (Nakashima, Iohara, Murakami) demonstrate high citation impacts, suggesting a strong research presence and influence in their respective fields.

Top Occurred Keywords

Table 6 displays the details of the top 20 most frequently occurring keywords. Occurrences mean the number of times each keyword has been used by the authors in the dataset and Total Link Strength shows the extent of the strength of connections or relevance of each keyword within the context. The keyword “Pulpectomy” has been the most repeatedly occurring keyword, with 192 occurrences and a total link strength of 161. This advocates it as a dominant theme in the research. The keyword “Primary teeth” follows with 84 occurrences and a link strength of 85, representing its prominence in pediatric dentistry. “Pulpotomy” occupied the third rank with 82 occurrences and a relatively high link strength of 131, indicating both frequent use and strong influences in research consultations. Partial pulpotomy (52 occurrences) and mineral trioxide aggregate occurred 52 and 47 times, respectively, and were also momentous, showing a focus on explicit techniques and materials in endodontics.

**Table 6:** Top 20 keywords

Serial No.	Keyword	Occurrences	Total Link Strength
1.	Pulpectomy	192	161
2.	Primary Teeth	84	85
3.	Pulpotomy	82	131
4.	Partial Pulpotomy	52	59
5.	Mineral Trioxide Aggregate	47	88
6.	Calcium Hydroxide	46	79
7.	Endodontics	40	40
8.	Vital Pulp Therapy	35	54
9.	Primary Molars	29	25
10.	Dental Pulp	27	21
11.	Pulpitis	27	44
12.	Pulp Capping	25	47
13.	Dental Pulp Exposure	24	33
14.	Irreversible Pulpitis	24	29
15.	Direct Pulp Capping	21	34
16.	Dental Caries	19	29
17.	Pulp Regeneration	17	8
18.	Root Canal Treatment	17	18
19.	MTA	15	22
20.	Pediatric Dentistry	14	17

Ten Most Cited Papers

The top 10 cited papers are shown in Table 7, which gained an overall 2235 citations with an average of 223.50 cites/paper. These papers were published from 2007 to 2019 and consisted of six original research articles as well as four review articles. These papers were published in six journals and most of the papers (n=3) were published in International Endodontic Journal, followed by two each in Journal of Endodontics and Journal of Dental Research. All the papers were written in collaboration patterns varying the authorship patterns from two authors to 18 authors and the 60 authors contributed belonged to 12 countries. Lars Bjorndal of University of Copenhagen, Denmark produced three top cited papers, followed by 10 authors with two articles each. The authors from Denmark, England and United States contributed three papers each, while authors from five countries (France, Germany, Ireland, Japan and Sweden) contributed two papers each.

**Table 7:** Top 10 most cited papers

Serial No.	Title of the Article	Types of Paper	Total Citations	Citation Density by Year (Rank)
1.	Mineral trioxide aggregate and other bioactive endodontic cements: an updated overview - part I: vital pulp therapy [17].	Review	278	39.71 (2)
2.	European Society of Endodontology position statement: management of deep caries and the exposed pulp [18].	Article	266	44.33 (1)
3.	Regeneration by transplantation of dental pulp stem cells in pulpitis: a pilot clinical study [19].	Article	262	32.75 (3)
4.	Vital pulp therapy in vital permanent teeth with cariously exposed pulp: a systematic review [20].	Review	249	17.79 (5)
5.	Complete pulp regeneration after pulpectomy by transplantation of CD105+ stem cells with stromal cell-derived factor-1 [21].	Article	226	16.14 (6)
6.	Treatment of deep caries lesions in adults: randomized clinical trials comparing stepwise vs. direct complete excavation, and direct pulp capping vs. partial pulpotomy [22].	Article	214	14.27 (8)
7.	Pulp revascularization of immature dog teeth with apical periodontitis [23].	Article	204	11.33 (10)
8.	Management of deep caries and the exposed pulp [24].	Review	195	32.50 (4)
9.	Comparison of CaOH with MTA for direct pulp capping: a PBRN randomized clinical trial [25].	Article	172	14.33 (7)
10.	Incomplete caries removal: a systematic review and meta-analysis [26].	Review	169	14.08 (9)

Discussion

The bibliometric studies quantitatively analyzed the various features of scholarly publications such as periodic growth, citation impact, channels of publication, top authors, top countries, and institutions within a particular topic [27,28]. Bibliometric studies can support practitioners and academics navigate the large landscape of scholarly literature by highlighting trends, important publications, and developing fields of study [29]. The WoS is an extensive database that provides bibliographic and citation data of the quality publications on diverse academic disciplines, including dentistry. It is an important tool for bibliometric research because it indexes high-impact journals, books and conference proceedings [30].

The study examined the evolution of research published in pulpotomy over a span of 24 years (2000-2023) using a broad diagnostic bibliometric approach. The findings exhibit stimulating outcomes, as 738 papers were identified in WoS data and these papers gained an average of 18.74 citations per paper. The first half of the period (2000-2011) had the highest ratio of citations (30.71 cites/paper), and the papers produced in the latter half of the period (2012-2023) had the lowest citation impact because the citation rates often increase gradually. Most of the papers (82.12%) were published in the latter half; the increased quantity of publications validated an increase in the number of research projects and institutions as well as growth in the research community. In line with this finding, a study examining the literature on vital pulp therapy from 1949 to 2020 reported more than 60% of papers after 2012 [14].

The analysis of LoEs shows that most of the papers dealt with LoE-IV but the papers with the high-quality evidence (LoE-I) gained the highest citation impact. Jamjoom et al. examined the LoEs on papers published in Saudi Dental Journal and it was found that more than half of the papers (51.53%) had lower LoE-IV and V [31]. In our study, 46.47% of LoEs marked in LoE-IV and V. Our findings also reported that the closed-accessed and papers published in dental journals received more attention from the scholarly community as compared to open-accessed and non-dental journal papers.

Papers published in high-impact factor journals are more likely to be cited in subsequent research, as indicated by their tendency to correlate with larger average citation impact. The variations in the citation impacts demonstrate the diversity in the views and applications of various publications within their respective domains. Journals that have smaller influence on citations should look into ways to boost exposure and interaction, such as promoting papers or concentrating on new developments in their industries [32]. In our study, 213 different sources have been identified and 40% (n=296) of the papers published in top 10 journals. Journal of Endodontics leads, followed by International Endodontics Journal and Journal of Clinical Pediatric Dentistry. Journal of Dental Research has the highest impact factor (5.7) and a very high citation impact (58.36), indicating that its papers are extensively cited qualified to their volume, followed by International Endodontics Journal (5.4) also displaying strong citation impact (42.29 cites/paper), reflecting its impact in the field. The information indicates that impact factors and citation metrics are useful markers of a journal's reputation in the academic community and emphasizes the significance of both quantity and quality in scholarly publishing. A bibliometric study on endodontic therapy in primary teeth revealed that Pediatric Dentistry Journal and International Endodontic Journal were found most preferred sources [13]. A study focused on vital pulp therapy exposed that most of the papers were published in Journal of Endodontics, followed by Pediatric Dentistry and International Endodontic Journal [14].

According to our analysis, India publishes a large number of papers, but their average citation impact is lower, suggesting that there may be problems with the visibility or applicability of their research. On the other hand, even though England and France publish fewer papers, they gain higher citation impacts, indicating that the academic community values or recognizes their work. The United States and Brazil exhibit a robust mix of overall citations and citation impact, indicating that they are not just generating substantial amounts of research but also highly referenced work. This analysis emphasizes the value of both quantity and quality in research, as well as the disparities in the impact and level of worldwide acknowledgment of research outputs among countries. A study on 100 most cited articles on endodontic therapy in primary teeth showed that the United States produced 21 articles followed by Brazil and Turkey [13]. Another study examined the scholarly literature on vital pulp therapy and reported that most of papers contributed by the United States, Iran and Brazil [14].

Evaluating the institutional research output has gained significance, because rankings can impact organization's reputation and even in fundraising capacity [33]. Our study reveals that Aichi Gakuin University and the National Center for Geriatrics and Gerontology are two examples of institutions that have highest citation impacts even though they publish fewer publications. This emphasizes the value of high-quality research above quantity. They also demonstrate strong scientific influence and a well-established academic culture in related subjects. The finding shows that the quantity of papers published has always equate to a high citation impact. Author such as Murakami show that fewer publications do not always equal greater influence. With numerous authors displaying significant citation impacts, Japan stands out as a country with a strong focus on pulpotomy and related subjects as well as a robust research culture. Some authors publish more work with relatively less recognition, while others obtain significant citation impacts with fewer works. Understanding possible mentorship opportunities, partnership opportunities, and areas where research exposure may be improved could all benefit from obtaining this information.

In bibliometrics, keyword analysis has been a prominent field of interest. The identification of novel and valuable bibliometric indicators using keyword occurrence plays an important role in encouraging the further development of this field [34]. High occurrence keywords typically have stronger links, suggesting that they are not only well-liked but also strongly associated with other pertinent ideas in the field. There are several reasons for utilizing key terms (i) primarily, three to five essential concepts in an author's work are encapsulated by keywords; (ii) secondly, to uncover prevalent study topics from both historical and contemporary contexts by keyword analysis (iii) finally, likelihood of a publication being cited increases with the presence of specific associated keywords [35].

In the present study, papers published in dental journals significantly (p=0.009) performed better than those in non-dental journals, while the proportion of citation difference between open and subscription was also significant (p<0.00). Similar results have been obtained in other bibliometric studies [27,35].

According to the analysis, the majority of the research in this dataset is focused on keywords associated with certain operations (such as pulpectomy and pulpotomy) and materials (such as mineral trioxide aggregate). Although they might not have got as much momentum in terms of frequency and link strength as they have, yet keywords like “pulp regeneration” found emerging area of interest. The information demonstrates the hot subjects in dental research, especially endodontics and pediatric dentistry. The intensity of the links between these terms reveals their relevance and interconnectedness, pointing to a clearly defined landscape of research goals. Recognizing these patterns can assist researchers in determining crucial subjects for upcoming research projects and team efforts.

Limitations

This study has multiple shortcomings. It is possible that by focusing on particular keywords to identify scientific literature, relevant studies that did not include those keywords were missed. An extensive comprehension of the pulpotomy research output could be attained by doing more studies with a more inclusive term. Then, we added every kind of document that we could find on WoS. On the other hand, future researchers might exclusively incorporate peer-reviewed literature, such as research and review articles. For a more thorough and comprehensive understanding of the topic, publication records in additional databases such as PubMed and Scopus as well as the incorporation of gray literature will be more advantageous for the future studies. Furthermore, with the exception of the clinical and non-clinical data and LoEs, the study was primarily quantitative and concentrated more on numerical data. Thus, it will be beneficial to do supplementary research assessing the subject dispersion about pulpotomy.

## Conclusions

This analysis shows significant variations in research output and citation impact among different institutions. High citation impact indicates influential work, while higher output with lower impact suggests potential areas for improvement in research visibility and relevance. Institutions may benefit from strategies that enhance the dissemination and recognition of their research to improve citation outcomes. Overall, this data can guide future research collaborations and highlight successful research strategies.
